# Prevalence and phylogeny of *Chlamydiae* and hemotropic mycoplasma species in captive and free-living bats

**DOI:** 10.1186/s12866-020-01872-x

**Published:** 2020-06-26

**Authors:** Janine Fritschi, Hanna Marti, Helena M. B. Seth-Smith, Sébastien Aeby, Gilbert Greub, Marina L. Meli, Regina Hofmann-Lehmann, Kristin Mühldorfer, Nadine Stokar-Regenscheit, Danja Wiederkehr, Paola Pilo, Peggy Rüegg- Van Den Broek, Nicole Borel

**Affiliations:** 1 Institute of Veterinary Pathology, Vetsuisse Faculty, University of Zurich, Zurich, Switzerland; 2 Center for Clinical Studies, Vetsuisse Faculty, University of Zurich, Zurich, Switzerland; 3 Institute of Microbiology, University of Lausanne, Zurich, Switzerland; 4 Clinical Laboratory, Department for Clinical Diagnostics and Services, Vetsuisse Faculty, University of Zurich, Zurich, Switzerland; 5 Department of Wildlife Diseases, Leibniz Institute for Zoo and Wildlife Research, Berlin, Germany; 6 Institute of Animal Pathology, Vetsuisse-Faculty, University of Bern, Bern, Switzerland; 7 Bern University of Applied Sciences, School of Agricultural, Forest and Food Sciences, Zollikofen, Switzerland; 8 Institute of Veterinary Bacteriology, Vetsuisse Faculty, University of Bern, Bern, Switzerland; 9 Stiftung Papiliorama, Kerzers, Switzerland

**Keywords:** *Chlamydiales*, Hemotropic mycoplasmas, Hemoplasmas, Bats, DNA, qPCR

## Abstract

**Background:**

Bats are hosts for a variety of microorganisms, however, little is known about the presence of *Chlamydiales* and hemotropic mycoplasmas. This study investigated 475 captive and free-living bats from Switzerland, Germany, and Costa Rica for *Chlamydiales* and hemotropic mycoplasmas by PCR to determine the prevalence and phylogeny of these organisms.

**Results:**

Screening for *Chlamydiales* resulted in a total prevalence of 31.4%. Positive samples originated from captive and free-living bats from all three countries. Sequencing of 15 samples allowed the detection of two phylogenetically distinct groups. These groups share sequence identities to *Chlamydiaceae*, and to *Chlamydia*-like organisms including *Rhabdochlamydiaceae* and unclassified *Chlamydiales* from environmental samples, respectively.

PCR analysis for the presence of hemotropic mycoplasmas resulted in a total prevalence of 0.7%, comprising free-living bats from Germany and Costa Rica. Phylogenetic analysis revealed three sequences related to other unidentified mycoplasmas found in vampire bats and Chilean bats.

**Conclusions:**

Bats can harbor *Chlamydiales* and hemotropic mycoplasmas and the newly described sequences in this study indicate that the diversity of these bacteria in bats is much larger than previously thought. Both, *Chlamydiales* and hemotropic mycoplasmas are not restricted to certain bat species or countries and captive and free-living bats can be colonized. In conclusion, bats represent another potential host or vector for novel, previously unidentified, *Chlamydiales* and hemotropic mycoplasmas.

## Background

Bats of the order *Chiroptera* are of increasing interest as potential reservoirs and vectors of pathogens. They possess unique characteristics among mammals, such as the ability to fly. Their extensive mobility, combined with their roost plasticity, nesting behavior and broad food range allows transport of pathogens to many different animal species in various locations [1]. It has been shown that bats are hosts for a multitude of different microorganisms that include viruses, bacteria, parasites and fungi. Several of these infectious agents are common to humans and domestic animals. The pathogenic potential of some bacterial species has been confirmed for bats, but the knowledge regarding the impact of such microorganisms on bat hosts is limited. In addition, knowledge on the natural microbiota of bats is scarce [2].

*Chlamydiae* are highly successful animal and human pathogens. Their taxonomic structure is composed of the order *Chlamydiales*, which consists of the nine families *Parachlamydiaceae*, *Waddliaceae*, *Simkaniaceae*, *Rhabdochlamydiaceae*, *Criblamydiaceae*, *Piscichlamydiaceae*, *Clavichlamydiaceae* and *Parilichlamydiaceae*, collectively referred to as *Chlamydia*-like organisms, and the *Chlamydiaceae* [3–5]. *Chlamydiae* are obligate intracellular bacteria with a biphasic developmental cycle. This cycle is characterized by the infectious but metabolically less active elementary body, which infects susceptible host cells, and the intracellular reticulate body, which undergoes binary fission [6]. As an inclusion fills with progeny, the reticulate bodies condense back into elementary bodies and are released by host cell rupture or by fusion of the inclusion membrane with the host cell plasma membrane. *Chlamydiae* are disseminated by aerosol or contact, requiring no alternative vector [4].

In 2005 and 2015, two novel *Chlamydia*-like organisms, *Waddlia malaysiensis* and *Waddlia cocoyoc* were detected in fruit bats in Malaysia and Mexico, respectively [7, 8]. In 2016, members of the order *Chlamydiales* were detected in the fecal bacterial microbiota of Daubenton’s bats in Finland [9].

Hemotropic mycoplasmas (hemoplasmas) are considered to be emerging or re-emerging zoonotic pathogens. They are classified in the order *Mycoplasmatales*, family *Mycoplasmataceae* and genus *Mycoplasma*. They are small, pleomorphic bacteria without a cell wall and parasitize red blood cells [10, 11]. The transmission routes of hemoplasmas are not yet fully understood, but they are thought to be transmitted through blood, saliva and possibly also arthropods [10, 12–14]. Hemoplasmas are able to cause acute infectious anemia or various chronic diseases in farm animals [15, 16], wild animals [17, 18], pets [14] and humans [19]. The extent of clinical manifestations ranges from asymptomatic to life-threatening [10].

From 2014 to 2017, four studies to determine the prevalence of hemoplasmas in various bat species were conducted [20–23]. The prevalence ranged from 18.5% in Brazil to 96.8% in Spain and sequences indicating new species or new genotypes were identified [20–23].

The objective of this study was to investigate the prevalence and phylogenetic positioning of *Chlamydiae* and hemotropic mycoplasmas species in 475 captive and free-living bats from six families and 28 species from Switzerland, Germany, and Costa Rica (Table 1).

**Table 1:** Sampled bats categorized according to origin, family, species and number of animals

Origin		Family	Species	Number of Animals
Captive	Switzerland	*Phyllostomidae*	*Carollia perspicillata*	89
	Germany	*Phyllostomidae*	*Carollia perspicillata*	13
			*Glossophaga commissarisi*	2
			*Glossophaga soricina*	2
			*Phyllostomus discolor*	5
		*Pteropodidae*	*Eidolon helvum helvum*	4
			*Rousettus aegyptiacus*	1
		*Megadermatidae*	*Megaderma lyra*	1
Free-living	Switzerland	*Vespertilionidae*	*Eptesicus nilssonii*	1
			*Eptesicus sp.*	1
			*Hypsugo savii*	1
			*Myotis daubentonii*	2
			*Myotis myotis*	18
			*Myotis mystacinus*	8
			*Myotis nattereri*	2
			*Nyctalus leisleri*	2
			*Nyctalus noctula*	6
			*Pipistrellus kuhlii*	28
			*Pipistrellus nathusii*	18
			*Pipistrellus pipistrellus*	123
			*Pipistrellus pygmaeus*	4
			*Pipistrellus sp.*	47
			*Plecotus auritus*	4
			*Plecotus sp.*	3
			*Vespertilio murinus*	8
		*Rhinolophidae*	*Rhinolophus ferrumequinum*	2
			*Rhinolophus hipposideros*	2
		*Unknown*	*Unknown*	5
	Germany	*Vespertilionidae*	*Barbastella barbastellus*	2
			*Eptesicus nilssonii*	6
			*Eptesicus serotinus*	15
			*Nyctalus leisleri*	1
			*Nyctalus noctula*	16
			*Plecotus auritus*	7
			*Vespertilio murinus*	9
	Costa Rica	*Phyllostomidae*	*Artibeus watsoni*	1
			*Carollia perspicillata*	1
			*Glossophaga commissarisi*	13
		*Vespertilionidae*	*Rhogeessa io*	1
		*Emballonuridae*	*Saccopteryx bilineata*	1
**Total**				**475**

## Results

### Polymerase chain reaction (PCR) and sequencing results for *Chlamydiales*

**Fig. 1:**
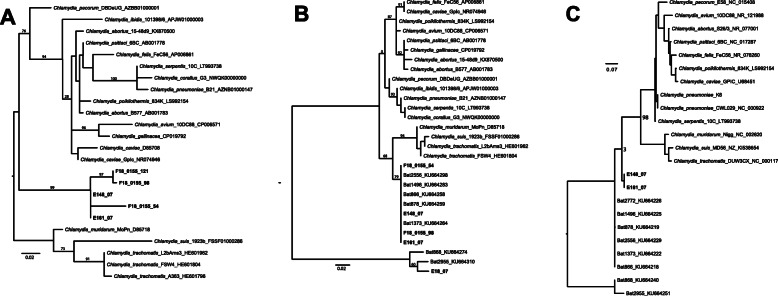
Phylogenetic trees based on partial sequences of the 16S and the 23S rRNA genes that show the relationships of the sequences obtained in this study and publicly available sequences of *Chlamydia* species and selected published sequences found in bats reflecting the phylogenetic relationship between known *Chlamydia* species based on nine genes [24]. **a** Phylogeny of chlamydial 16S rRNA gene, 284 bp covering V1 – V2, including all novel sequences and illustrating that they fall together within a novel clade. **b** Phylogeny of chlamydial 16S rRNA gene, 200 bp covering V3, relating available novel sequences to those previously found in bat samples and illustrating that these samples are closely related to previous bat samples over this region. **c**. Phylogeny of chlamydial 23S rRNA gene, 530 bp, relating available novel sequences to those previously found in bat samples and illustrating differences between the novel samples and previous bat samples across this region. Bat samples were selected from those published in Hokynar et al. [9] to reflect closely related samples and outgroup “*Rhabdochlamydiaceae*-like” samples. Bootstraps of 100 replicates are shown on key branches. Scale bar shows number of substitutions per site. Samples from this study are shown in bold

A total of 166/1021 DNA samples (16.3%) originating from 149/475 bats (31.4%) were positive for *Chlamydiales* DNA by real-time PCR. Of these 166 samples, 15 samples had a Ct value below 35.0 and were therefore sequenced, resulting in 17 consensus sequences comprising two to four sequences (Table 2). A BLASTn analysis and phylogenetic trees (Fig. 1) of these sequences revealed two groups and suggest that these sequences may represent two novel species-level lineages.

**Table 2:** Sequencing results of *Chlamydiales* and hemotropic mycoplasmas positive bats including the top BLASTn hit (query cover 98–100%) and identity results

Sample ID	Organ source	Bat species & origin	Applied PCR (Product size)	Top BLASTn hit (Accession No.)	Identity
52	Spleen	*Carollia perspicillata* (Switzerland, captive)	16S-pan-PCR (114/200 bp)	Uncultured *Chlamydiales* (MH119787.1)^b^	99.1%
95	Liver	*Carollia perspicillata* (Switzerland, captive)	16S-pan-PCR (181/200 bp)	Uncultured *Chlamydiales* (JX083073.1)^b^	91.7%
F18–0155.54	FFPE	*Myotis myotis* (Switzerland, free-living)	16S-IGF/IGR-PCR + 16S-pan-PCR (475/478 bp)	*Chlamydia pecorum* (AB001775.1)^a^	93.1%
F18–0155.98	FFPE	*Pipistrellus pipistrellus* (Switzerland, free-living)	16S-IGF/IGR-PCR + 16S-pan-PCR (510/478 bp)	*Chlamydophila pecorum* (D85716.1)^a^	93.1%
F18–0155.121	FFPE	*Pipistrellus* sp. (Switzerland, free-living)	16S-IGF/IGR-PCR + 16S-pan-PCR (281/478 bp)	Uncultured *Chlamydiales* (MK112598.1)^b^	92.3%
F18–0155.130	FFPE	*Pipistrellus pipistrellus* (Switzerland, free-living)	16S-pan-PCR (104/200 bp)	Uncultured *Chlamydiales* (MF440154.1)^b^	100.0%
F18–0155.145	FFPE	*Pipistrellus pipistrellus* (Switzerland, free-living)	16S-pan-PCR (112/200 bp)	Uncultured bacterium (LC336010.1)^b^	99.1%
E 4/08	Intestine	*Glossophaga soricina* (Germany, captive)	16S-pan-PCR (155/200 bp)	Uncultured bacterium (AB618438.1)^b^	97.4%
E 6/08	Intestine	*Glossophaga soricina* (Germany, captive)	16S-pan-PCR (159/200 bp)	Uncultured bacterium (KY692835.1)^b^	96.9%
E 148/07	Intestine	*Nyctalus noctula* (Germany, free-living)	16S-IGF/IGR-PCR + 16S-pan-PCR (460/478 bp)	*Chlamydia pecorum* (AB001775.1)^a^	93.5%
			23SIG-PCR (519/700 bp)	Uncultured *Chlamydiales* (KU664232.1)^a^	95.6%
E 155/07	Intestine	*Nyctalus noctula* (Germany, free-living)	16S-pan-PCR (95/200 bp)	*Chlamydia psittaci* (CP047319.1)^a^	95.8%
E 161/07	Intestine	*Eptesicus serotinus* (Germany, free-living)	16S-IGF/IGR-PCR + 16S-pan-PCR (506/478 bp)	*Chlamydia pecorum* (AB001775.1)^a^	94.1%
			23SIG-PCR (522/700 bp)	Uncultured *Chlamydiales* (KU664232.1)^a^	95.6%
E 179/07	Intestine	*Nyctalus noctula* (Germany, free-living)	16S-pan-PCR (201/200 bp)	*Chlamydiales* bacterium (MF620054.1)^a^	97.4%
E 197/07	Intestine	*Vespertilio murinus* (Germany, free-living)	16S-pan-PCR (158/200 bp)	Uncultured *Chlamydiales* (HQ721227.1)^b^	99.4%
E 18/07	Intestine	*Rhogeessa io* (Costa Rica, free-living)	16S-IGF/IGR-PCR (269/278 bp)	*Rhabdochlamydiaceae* bacterium (MF620051.1)^b^	96.3%
**Hemotropic Mycoplasmas**					
**Sample ID**	**Organ source**	**Bat species & origin**	**Method**	**Top BLASTn hit (Accession No.)**	**Identity**
E 173/07	Spleen	*Nyctalus noctula* (Germany, free-living)	HemMycop (41/938 + 322/1420)-PCR (1151/1901 bp)	Uncultured *Mycoplasma* sp. (MK295631.1)	97.3%
E 190/07	Spleen	*Vespertilio murinus* (Germany, free-living)	HemMycop (41/938 + 322/1420)-PCR (1196/1901 bp)	Uncultured *Mycoplasma* sp. (MK295631.1)	98.6%
E 70/06	Spleen	*Glossophaga commissarisi* (Costa Rica, free-living)	HemMycop41/938-PCR (774/871 bp)	Uncultured *Mycoplasma* sp. (KY932722.1)	96.3%
**Others**					
F18–0155.55	FFPE	*Pipistrellus kuhlii* (Switzerland, free-living)	HemMycop (41/938 + 322/1420)-PCR (741/1901 bp)	Uncultured bacterium (MK372594.1)	99.4%
F18–0155.63	FFPE	*Pipistrellus pipistrellus* (Switzerland, free-living)	HemMycop41/938-PCR (743/871 bp)	Uncultured bacterium (MK372594.1)	99.5%
F18–0155.70	FFPE	*Nyctalus noctula* (Switzerland, free-living)	HemMycop (41/938 + 322/1420)-PCR (1182/1901 bp)	Uncultured *Sphingomonadaceae* (EF019656.1)	92.8%
F18–0155.99	FFPE	*Pipistrellus kuhlii* (Switzerland, free-living)	HemMycop (41/938 + 322/1420)-PCR (886/1901 bp)	Uncultured bacterium (MK372594.1)	99.5%
F18–0155.102	FFPE	*Pipistrellus kuhlii* (Switzerland, free-living)	HemMycop (41/938 + 322/1420)-PCR (503/1901 bp)	*Bradyrhizobium* sp. (MT102777.1)	98.8%
F18–0155.110	FFPE	*Myotis daubentonii* (Switzerland, free-living)	HemMycop (41/938 + 322/1420)-PCR (1266/1901 bp)	*Altererythrobacter* sp. (MK574878.1)	92.6%
F18–0155.113	FFPE	*Nyctalus noctula* (Switzerland, free-living)	HemMycop (41/938 + 322/1420)-PCR (748/1901 bp)	*Bradyrhizobium* sp. (MG588424.1)	89.3%
F18–0155.116	FFPE	*Pipistrellus* sp. (Switzerland, free-living)	HemMycop (41/938 + 322/1420)-PCR (950/1901 bp)	*Bradyrhizobium* sp. (MK638083.1)	99.9%
E 196/07	Spleen	*Eptesicus nilssonii* (Germany, free-living)	HemMycop (41/938 + 322/1420)-PCR (951/1901 bp)	Uncultured bacterium (MK372594.1)	98.9%

^a^Chlamydial sequences assigned to group 1

^b^Chlamydial sequences assigned to group 2

Group one contained eight consensus sequences from six free-living bats (F18–0155.54, F18–0155.98, E 148/07, E 155/07, E 161/07 and E 179/07) originating from Switzerland and Germany that carry bacteria belonging to the *Chlamydiaceae*-family according to the classification scheme by Pillonel et al. [24] and phylogenetic analysis. The best BLASTn hits of the sequences obtained by the 16S-pan-PCR alone (95–201 base pair, bp) were primarily with sequences from uncultured *Chlamydiales* bacterium clone 1778 (GenBank accession number KU664271.1), from *Chlamydiales* bacterium isolate CL gt1 (GenBank accession number MF620054.1) and from *Chlamydia psittaci* strain AMK (GenBank accession number CP047319.1). The best BLASTn hits for consensus sequences obtained from both the 16S-IGF/IGR-PCR and the 16S-pan-PCR (size: 281–475 bp) was *Chlamydia pecorum* strain B0-Maeda (GenBank accession number AB001775.1) and *Chlamydophila pecorum* strain Ov/IPA (GenBank accession number D85716.1). Within this group phylogenetic analysis of 16S ribosomal ribonucleid acid (16S rRNA) and 23S ribosomal ribonucleid acid (23S rRNA) gene sequences shows that they cluster and belong to a novel branch most closely related to the *Chlamydia* genus (Table 2, Fig. 1a-c). Further published sequences from bats are also closely related to these [9] (Fig. 1b,c).

Group two contained 9 sequences from bacteria within four captive and five free-living bats (Sample IDs 52, 95, F18–0155.121, F18–0155.130, F18–0155.145, E 4/08, E 6/08, E 148/07, E 161/07, E197/07 and E 18/07) originating from Switzerland, Germany, and Costa Rica. They are closely related to sequences from uncultured *Chlamydiales* and from the *Rhabdochlamydiaceae* bacterium isolate P gt1 (GenBank accession number MF620051.1) (Table 2). Not all sequences gave clearly interpretable traces: one 16S rRNA sequence gave sufficient quality for phylogenetic analysis and shows this relationship to previously characterized sequences from bats [9] (Fig. 1b).

### PCR and sequencing results for hemotropic mycoplasmas

**Fig. 2:**
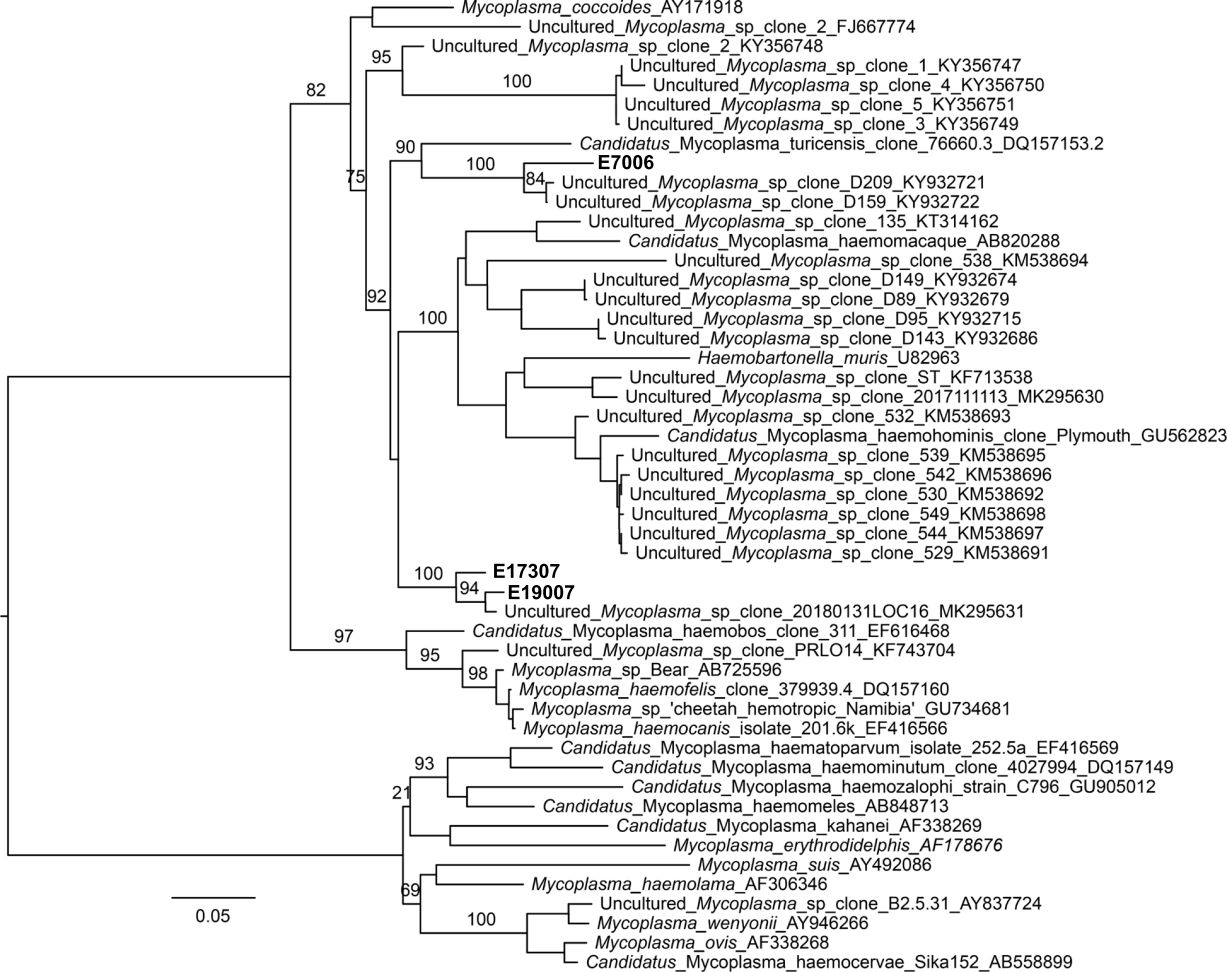
Phylogenetic tree showing the relationships of three sequences from this study and publicly available sequences of *Mycoplasma* species. Phylogenetic tree based on sequences obtained from PCR products of the 16S rRNA gene of hemotropic mycoplasmas, 1252 bp (minimum 824 bp). Bootstraps of 100 replicates are shown on key branches. Scale bar shows number of substitutions per site. Samples from this study are shown in bold

A total of 15/475 DNA samples (3.2%) originating from 15/462 bats (3.3%) tested positive or questionably positive for hemotropic mycoplasmal DNA by at least one of the two real-time PCRs used for screening. All 15 samples were also positive by conventional 16S rRNA gene PCR and were sent for sequencing, which resulted in 12 sequences that were readable in both directions. BLAST analysis revealed that three sequences fall in two clades that were closely related to either uncultured *Mycoplasma* sp. clone 20180131LOC1.16 or clone D159 (GenBank accession numbers MK295631.1 and KY932722.1) with a sequence identity of 96.3 to 98.6% (Table 2). The phylogenetic tree constructed from these 16S rRNA gene sequences (Fig. 2) shows that they are closely related to previously published sequences from vampire bat samples [23] and sequences from Chilean bat samples [25].

The remaining nine sequences were closely related to Proteobacteria by BLAST analysis (Table 2 “Others”).

## Discussion

Bats are known to be important vectors and reservoirs for a multitude of different microorganisms such as lyssaviruses, henipaviruses [2], *Leptospira* sp. [26] and *Bartonella* spp. [27], but the knowledge regarding their potential role as carriers of other clinically significant pathogens or less common bacteria is limited. To date, the only *Chlamydiales* identified in bats are two *Chlamydia*-like organisms: *Waddlia malaysiensis* [7] and *Waddlia cocoyoc* [8], isolated from fruit bats in Malaysia and Mexico, respectively, and members of the order *Chlamydiales*, isolated from a common bat species (*Myotis daubentonii*) in Finland [9].

### PCR and sequencing results for *Chlamydiales*

Phylogenetic analysis of the sequences obtained in the present study resulted in the formation of two groups: *Chlamydiaceae*-like and *Chlamydiales*-like. Separation in these groups had been previously described in the study from Hokynar et al. [9]. However, a precise classification according to the scheme from Pillonel et al. [24] was not possible based on short amplicons retrieved in this study and also because only 16S and 23S rRNA gene products were sequenced. The phylogenetic trees showed that five samples from group one (Sample IDs F18–0155.54, F18–0155.98, E 148/07, E 161/07 and E 18/07) are closely related to the samples from Hokynar et al. and four of them (Sample IDs F18–0155.54, F18–0155.98, E 148/07 and E 161/07) form a novel clade within the *Chlamydiaceae* family. These five samples originated from free-living bats from Switzerland and Germany belonging to the bat species *Myotis myotis*, *Pipistrellus pipistrellus*, *Nyctalus noctula* and *Eptesicus serotinus*. Therefore, it can be said that this clade first discovered in Finland occurs also in other parts of Europe and colonizes various species of free-living insectivorous bats. In the present study, these *Chlamydiae* were detected directly in the inner organs; therefore, these bats might actually act as a host, meaning that the *Chlamydiae* are not merely prey-borne, as hypothesized by Hokynar et al. The latter study only investigated bat feces and insect material, inner organs were not available. Although the present study indicates the presence of chlamydial DNA in inner organs, such as the intestine, detection of replicating chlamydial organisms could not be assessed; Detection of replication would have required the attempt of isolation, which was hampered by the unavailability of fresh frozen tissue material. Chlamydial DNA might originate from the diet or represent colonization and/or real infection [9]. If the latter holds true, this would suggest that the different chlamydial species documented in bats might have a pathogenic role for the bats or that these bats act as vectors.

In a study by Hornok et al. [28], 196 individual and 25 pooled fecal samples collected from 19 bat species from Hungary and the Netherlands were investigated for the presence of chlamydial DNA; they all tested negative indicating that the prevalence of *Chlamydiae* in bats is highly variable in studies from neighboring countries. This observation suggests that, although this new *Chlamydiae* clade may occur in some parts of Europe, colonization or infection of bats is influenced by factors that remain to be defined. Methodological differences may play a part of these factors.

Also a *Rhabdochlamydiaceae*-like sequence was identified in a sample (E 18/07) taken from a free-living bat from Costa Rica, which formed another novel clade with bat samples from Hokynar et al. [9] in the phylogenetic tree (E18_07). The remaining bat samples, similar to chlamydial sequences from environmental samples retrieved from the NCBI database, originated from captive and free-living bats from Switzerland and Germany. *Chlamydia*-like organisms are commonly present in water and inside amoebae [29–31], and have also been observed in ticks [32, 33] and fleas [34]. Thus, it is difficult to guess, which of these chlamydial putative vectors (amoebae or ticks) is the more likely at play as reservoir and vector.

In previous studies from Chua et al. [7] and Piérle et al. [8], two novel *Chlamydia*-like organisms belonging to the *Waddliaceae* family and named *Waddlia malaysiensis* and *Waddlia cocoyoc* were isolated from fruit bats from Malaysia and the municipality Cocoyoc in Mexico. These regions have a tropical climate, whereas a temperate climate predominates in Switzerland and Germany. In this study, bats from a tropical climate (Costa Rica) were analysed as well, but compared to the two other studies (n = 206 and n = 38) our sample size from Costa Rica (n = 17) was small. Another reason for the absence of *Waddliaceae* in bats of this study might the different sample type. *Waddlia malaysiensis* was first detected in urine samples from bats [7] but we did not investigate urine in this study. *Waddlia cocoyoc* was detected in DNA from the skin and in infected Vero and BHK 21 cells, but also caused severe lesions in lungs and spleen [8]. Therefore, it can be assumed that bacterial DNA would have been detected in these two organs if bacteria were present. The two isolates from Mexico and Malaysia might be bound to the regions and/or the climate.

In this study as well as in the studies from Hokynar et al. and Chua et al., only short fragments of the 16S rRNA and 23S rRNA genes were amplified, as many of the samples in the present study were only available as FFPE samples because the bats had died in the wild and were subsequently collected and sampled, therefore preventing detailed classification.

### PCR and sequencing results for *Mycoplasmatales*

In the current study, three sequences were closely related to uncultured *Mycoplasma* sp. and nine sequences were closely related to *Proteobacteria*. The amplification of *Proteobacteria*-like sequences might be the result of unspecific primer binding yielding false-positive PCR amplification.

The three sequences related to uncultured *Mycoplasma* sp. originated from the samples of free-living bats from Germany and Costa Rica belonging to the bat species *Nyctalus noctula*, *Vespertilio murinus* and *Glossophaga commissarisi*. Consequently, different species from the bat families *Vespertilionidae* and *Phyllostomidae* can harbor hemotropic mycoplasmas. The presence of hemoplasmal DNA could be only confirmed in free-living bats, which is in agreement with the presumed transmission pathways of hemotropic mycoplasmas through blood, saliva and arthropods [10, 12–14]. The captive bats were isolated from the environment and thus arthropod contact was at least partially excluded or reduced. Moreover, none of them were sanguivory bats and therefore do not bite other animals living in the enclosure. Consequently, transmission by blood, saliva and arthropods is very unlikely in captive bats.

The phylogenetic tree shows a close relationship between the sequences of the German bats among each other (E17307 and E19007) and a sequence from a Chilean bat [25]. The sequence from Costa Rican bat (E7006), on the other hand, was more closely related to sequences of vampire bats from Peru and Belize [23]. Vampire bats and the Commissaris’s long-tongued bat (*Glossophaga commissarisi*) belong to the family of leaf-nosed bats and Peru, Belize and Costa Rica are geographically close to each other strengthening the high degree of relationship in the phylogenetic tree.

In other studies by Ikeda et al. [20], Mascarelli et al. [21], Millan et al. [22] and Volokhov et al. [23], a much higher mycoplasmal prevalence (18.5 to 97%) was found than in the present study (0.7%), despite partially similar numbers of samples. A similar prevalence (1.8%) was only reported in the study by Hornok et al. [28]. Millan et al. suggested that subclinical infections with mycoplasmas in bats are common. This may be related to distinct climatic conditions and thus different arthropod activity, which are suspected to act as vectors of hemotropic mycoplasmas. In all studies, only fragments of the 16S rRNA encoding gene were sequenced and then combined into contigs to optimize the chances of getting a larger 16S rRNA encoding gene sequence for further analysis. This study gives insight into the diversity of hemotropic mycoplasmas in different bat species from three geographical regions, although a more detailed description of the sequences and a more precise taxonomical classification were not possible.

Bats belong to the superorder of the *Laurasiatheria* and therefore are more closely related to carnivores, even-toed and odd-toed ungulates, than to rodents and primates, which belong to the superorder of the *Euarchontoglires*. While the relationship of other hemotropic mycoplasmas represented in the phylogenetic tree (Fig. 2) roughly corresponds to that of the mammalian classification, *Mycoplasma* of bats are the only ones to classify quite differently. This is likely due to the high resistance of bats to pathogenic microorganisms, somehow similar to some rodents (mice, rats). Moreover, this suggests that bats do not get infected by exposure to meat, but rather by exposure to water (and free-living amoebae) as well as to ectoparasites colonizing bats (such as *Spinturnix*). The latter hypothesis is supported for *Chlamydia*-like organisms by the recent work of Thiévent et al. that demonstrated the occurrence of *Chlamydia*-related bacteria in several *Spinturnix* species [35].

## Conclusions

*Chlamydiaceae*, *Chlamydia*-like organisms and hemotropic mycoplasmas have been identified in humans and various animal species but only a few studies investigated their presence in bats. This study assessed the prevalence and phylogeny of chlamydial and hemoplasmal DNA in captive and free-living bats from Switzerland, Germany and Costa Rica. Chlamydial and hemoplasmal sequences, similar to sequences obtained from bats and their prey investigated in other studies and from environmental samples, were identified. Newly described sequences indicate that the diversity of these bacteria in bats is broader than previously thought. Neither *Chlamydiales* nor hemotropic mycoplasmas are restricted to certain bat species or countries, and captive and free-living bats can be colonized.

## Methods

### Bat sampling and DNA extraction

A total number of 475 bats belonging to six bat families and 28 bat species were sampled (Table 1). Lungs, liver, intestines and spleen from 89 captive bats from Switzerland, 28 captive and 55 free-living bats from Germany, and 17 free-living bats from Costa Rica were investigated. Thereof, bat samples from Germany and Costa Rica were obtained from the Department of Wildlife Diseases, Leibniz Institute for Zoo and Wildlife Research, Berlin, Germany and samples from Switzerland were obtained from the Institute of Animal Pathology, Vetsuisse-Faculty, University of Bern, Switzerland, from the Stiftung Papiliorama, Kerzers, Switzerland and from Dr. Danja Wiederkehr, collection curator from the Swiss bat preservation organization. The intestines were selected based on a previous study [9], where *Chlamydiae* were detected in bat feces. For this study, jejunum was available from the captive bats of Switzerland. Small and large intestines from Swiss free-living bats, captive and free-living bats from Germany and free-living bats from Costa Rica were pooled for DNA extraction. The spleen was selected as target organ for hemotropic mycoplasma detection. Lung and liver were selected because these are common target organs for chlamydial infections in mammals and birds. Moreover, we were interested to investigate the systemic (hematogenous) spread of *Chlamydiae* in these organs.

DNA extraction from bat samples from Switzerland was performed using the DNeasy Blood and Tissue Kit #69506 (Qiagen, Hilden, Germany) following manufacturer’s instructions, whereas DNA extraction of the samples from Germany and Costa Rica was performed using either the NucleoSpin® DNA RapidLyse (lung, liver, spleen) or the NucleoSpin® Tissue Kit (intestine) (MACHEREY-NAGEL GmbH & Co. KG, Düren, Germany). Additionally, inner organs including lungs, liver, intestines and spleen, from 285 free-living bats from Switzerland were obtained either as FFPE blocks or fixed in 4% formalin. The DNA of the FFPE blocks was extracted directly, while the organs fixed in 4% formalin were first embedded in paraffin according to routine procedures. The DNA extraction was then performed using the QIAamp DNA FFPE Tissue Kit #56404 (Qiagen) following manufacturer’s instructions. DNA quantity and quality of all samples was evaluated by spectrophotometry with the Nanodrop-1000 (Witec AG, Luzern, Switzerland).

### PCR analysis for *Chlamydiales* DNA

A total of 1021 DNA samples was screened for the presence of *Chlamydiales* DNA using two different real-time PCRs targeting sequentially the 23S rRNA gene (*Chlamydiaceae* family-specific) and the 16S rRNA gene (pan-*Chlamydiales* order-specific).

The *Chlamydiaceae*-specific real-time PCR targeting the 23S rRNA gene (Chlam23S quantitative polymerase chain reaction, qPCR) [36] used primers Ch23S-F, Ch23S-R and probe Ch23S-p (Microsynth, Balgach, Switzerland) described by Ehricht et al. [37]. The internal amplification control eGFP amplified with primers eGFP-1-F, eGFP-10-R and probe eGFP-Hex (Microsynth) was added to each reaction [38]. The PCR was conducted on a Thermocycler 7500 Fast ABI (Thermo Fisher Scientific). All samples were tested in duplicate and samples with a cycle threshold of < 38 in duplicate PCR reactions were considered positive. Quantitation was performed using 10-fold dilutions (10^7^ copies to 10 copies/μL) of the *Chlamydia abortus* genomic DNA positive control (standard curve).

Samples positive in the *Chlamydiaceae* family-specific real-time PCR were then further analysed using three different conventional PCR protocols targeting partial sequences of the 16S or 23S rRNA gene. The conventional PCR targeting a 278-bp fragment of *Chlamydiales* 16S rRNA gene (16S-IGF/IGR-PCR) was performed using primers 16S-IGF and 16S-IGR [39, 40] (Microsynth) modified from Everett et al. [4]. Primers 16S-panCh-F and 16S-panCh-R (Microsynth) were applied for the conventional PCR targeting a 200-bp fragment of the *Chlamydiales* 16S rRNA gene (16S-pan-PCR) [32] and the conventional PCR targeting a 700 bp fragment of *Chlamydiales* 23S rRNA gene (23SIG-PCR) [4, 32, 36] included primers U23-F and 23SIG-R (Microsynth). For all three conventional PCRs, cycling was performed on a Biometra TRIO Thermal Cycler (Analytik Jena AG, Jena, Germany) and PCR products were analysed by gel-electrophoresis on a 1.5% agarose gel.

For the pan-*Chlamydiales* real-time PCR targeting a partial sequence of the 16S rRNA-encoding gene (16S-pan-qPCR) [41], primers 16S-panCh-F, 16S-panCh-R and probe 16S-panCh (Eurogentec, Seraing, Belgium) were applied in a StepOne Plus real-time PCR system (Thermo Fisher Scientific). All samples were tested in duplicate, and if a single replicate was positive (Ct ≤ 37), the corresponding sample was considered positive. Quantification was performed using a 10-fold-dilution of a plasmid control tested in duplicate, constructed with the sequence of interest based on the *Parachlamydia acanthamoebae* 16S rRNA encoding gene, cloned with the TOPO TA Cloning Kit for Subcloning with One Shot TOP10 chemically competent *E. coli* cells (Thermo Fisher Scientific). Molecular-biology-grade water was used as a negative control in all PCR reactions.

All PCR primers and probes, the targeted genes and amplicon sizes used in this study are summarized in Table 3. All reaction mix compositions and cycling conditions are shown in Table S1.

**Table 3:** Primers and probes used in the present study

PCR method*	Gene target & amplicon size	Name	Sequence (5′ – 3′)	Reference
Chlam23S-qPCR	23S rRNA, 111 bp	Ch23S-F	CTGAAACCAGTAGCTTATAAGCGGT	Ehricht et al. 2006 [37]
		Ch23S-R	ACCTCGCCGTTTAACTTAACTCC	
		Ch23S-p	6-FAM-CTCATCATGCAAAAGGCACGCCG-TAMRA	
23SIG-PCR	23S rRNA signature sequence, 700 bp	U23-F	GATGCCTTGGCATTGATAGGCGATGAAGGA	Everett et al. 1999 [4, 36]
		23SIG-R	TGGCTCATCATGCAAAAGGCA	
16S-IGF/IGR-PCR	16S rRNA, 278 bp	16S-IGF	GATGAGGCATGCAAGTCGAACG	Pospischil et al. 2012 [39]; Blumer et al. 2007 [40]
		16S-IGR	CCAGTGTTGGCGGTCAATCTCTC	
16S-pan-qPCR^a^ 16S-pan-PCR^b^	16S rRNA, 200 bp^a,b^	16S-panCh-F^a,b^	CCGCAACACTGGGACT	Lienard et al. 2011 [41]^a^, Hokynar et al. 2016^b^ [32]
		16S-panCh-R^a,b^	GGAGTTAGCCGGTGCTTCTTTAC	
		16S-panCh^a^	6-FAM-CTACGGGAGGCTGCAGTCGAGAATC-BHQ1	
	sequencing primers^a^	panFseq^a^	CCAACACTGGGACTGAGA	
		panRseq^a^	GCCGGTGCTTCTTTAC	
eGFP-qPCR	eGFP, 177 bp	eGFP-1-F	GACCACTACCAGCAGAACAC	Blumer et al. 2011 [38]
		eGFP-10-R	CTTGTACAGCTCGTCCATGC	
		eGFP-Hex	VIC-AGCACCCAGTCCGCCCTGAGCA-none	
Hemoplasma SYBR Green qPCR	16S rRNA	Mhae_sybr.359f	AGCAATACCATGTGAACGATGAA	Willi et al. 2009 [42]
		Mcocc_sybrF	AGCAATGCCATGTGAACGATGAA	
		Mhae_sybr.432r	TGGCACATAGTTTGCTGTCACTT	
		Cmhae_Sybr.493r	GCTGGCACATAGTTAGCTGTCACT	
Mhf-like qPCR	16S rRNA, 114 bp	Group_Mhf_fwd	GGAGCGGTGGAATGTGTAG	Tasker et al. 2010 [43]
		Group_Mhf_rev	GGGGTATCTAATCCCATTTGC	
		Group_Mhf_probe	6-FAM-TYAAGAACACCAGAGGCGAAGGCG-BHQ1	
CMmh-like qPCR	16S rRNA, 139 bp	Group_CMhm_fwd	GGGGCCAAGTCAAGTCATC	Tasker et al. 2010 [43]
		Group_CMhm_rev	GCGAATTGCAGCCTTTTATC	
		Group_CMhm_probe	YYE-TACCATTGTAGCACGTTYGCAGCCC-BHQ1	
HemMycop41/938-PCR	16S rRNA, 871 bp	HemMycop16S-41 s	GYATGCMTAAYACATGCAAGTCGARCG	Mascarelli et al. 2014 [21]
		HemMycop16S-938as	CTCCACCACTTGTTCAGGTCCCCGTC	
HemMycop322/1420-PCR	16S rRNA, 1030 bp	HemMycop16S-322 s	GCCCATATTCCTACGGGAAGCAGCAGT	Mascarelli et al. 2014 [21]
		HemMycop16S-1420as	GTTTGACGGGCGGTGTGTACAAGACC	

*conventional PCR (PCR), real-time PCR (qPCR)

^a & b^PCRs performed according to the respective labeled reference, using the respective labeled primers

### PCR analysis for hemotropic mycoplasma DNA

All spleen and FFPE block samples (n = 475) from 462 bats were screened for the presence of hemoplasma DNA using a universal hemotropic mycoplasma-specific SYBR Green real-time PCR (Hemoplasma SYBR Green qPCR) targeting the 16S rRNA encoding gene, including primers Mhae_sybr.359f, Mcocc_sybrF, Mhae_sybr.432r and Cmhae_Sybr.493r (Microsynth) [42]. Samples within the same melting temperature range as the positive controls were considered positive.

Samples positive by SYBR Green real-time PCR were then further analysed by using real-time PCRs specific for *M. haemofelis*-like and ‘*Candidatus* M. haemominutum’-like organisms and two conventional PCRs targeting an 871-bp and 1030-bp region of the 16S rRNA encoding gene.

The *M. haemofelis*-like-specific real-time PCR (Mhf-like qPCR) targeting a 114-bp fragment of the 16S rRNA gene was performed using primers Group_Mhf_fwd and Group_Mhf_rev and probe Group_Mhf_probe (Microsynth) [43].

The ‘*Candidatus* M. haemominutum’-like-specific real-time PCR (CMhm-like qPCR) targeting a 139-bp fragment of the 16S rRNA gene included primers Group_CMhm_fwd and Group_CMhm_rev and probe Group_CMhm-probe (Microsynth) [43].

All three real-time PCRs were conducted on a Thermocycler 7500-Fast ABI. Molecular-biology-grade water was used as a negative PCR control and DNA of *Mycoplasma haemofelis*, ‘*Candidatus* Mycoplasma haemominutum’, ‘*Candidatus* Mycoplasma turicensis’ and ‘*Candidatus* Mycoplasma haematoparvum’ was used as a positive PCR control.

The conventional PCR targeting an 871-bp fragment of hemoplasmal 16S rRNA encoding gene (HemMycop41/938-PCR) was performed using primers HemMycop16S-41 s and HemMycop16S-938as (Microsynth). Primers HemMycop16S-322 s and HemMycop16S-1420as (Microsynth) were applied for the conventional PCR targeting a 1030-bp fragment of the hemoplasmal 16S rRNA encoding gene (HemMycop322/1420-PCR) [21]. Molecular-biology-grade water was used as a negative PCR control and DNA from both *Mycoplasma wenyonii* and *Mycoplasma haemocanis* were used as positive PCR controls. Cycling was performed on a Biometra T-personal Thermal Cycler (Biolabo Scientific Instruments, Châtel-Saint-Denis, Switzerland) and PCR products were analysed by gel-electrophoresis on a 1.5% agarose gel.

All PCR primers and probes, the targeted genes and amplicon sizes used in this study are summarized in Table 3. All reaction mix compositions and cycling conditions are shown in Table S1.

### Sequencing and analysing of *Chlamydiales*- and Hemoplasma-positive PCR products

For sequencing, amplicons of samples positive by conventional PCRs 16S-IGF/IGR-PCR, 16S-pan-PCR, 23SIG-PCR, HemMycop41/938-PCR or HemMycop322/1420-PCR were purified using the GeneJET PCR Purification Kit (ThermoFisher Scientific) or the GeneJET Gel Extraction Kit (ThermoFisher Scientific) according to manufacturer’s instructions. The forward and reverse strands of the PCR products were sequenced using the respective primers of the positive PCR reaction. Microsynth performed all sequencing reactions using Sanger sequencing.

Amplicons of samples positive by 16S-pan-qPCR with a Ct value ≤35.0 were a) either purified using an MSB Spin PCRapace kit (Invitek, Berlin, Germany) with a subsequent PCR reaction using a BigDye Terminator v1.1 cycle sequencing kit (Applied Biosystems) [41] or b) purified and sequenced by Microsynth using specifically designed inner primers panFseq and panRseq.

Sequence traces were visualized and assembled in CLC Genomics Workbench v10.1.1; assemblies were compared to known sequences in the NCBI database by BLAST analysis. Phylogenetic analyses on the assemblies and related database sequences were performed using muscle with default parameters in Seaview (44) with manual correction where necessary to create alignments, and using PhyML in Seaview with default parameters including GTR model, and 100 bootstrap replicates, to create phylogenetic trees.

## Supplementary information

**Additional file 1: Supplementary Table 1**. Reaction Mix compositions and cycling conditions of PCR methods used in this study.

## Data Availability

The sequences obtained in this study were deposited in the GenBank under accession numbers LR699022.1, LR699021.1, LR699020.1, LR584972.1, LR584971.1, LR584970.1; LR584969.1, LR584968.1, LR584967.1, LR584966.1, LR584965.1, released under the project 546195: https://www.ncbi.nlm.nih.gov/nuccore?term=546195%5BBioProject%5D

## Abbreviations

16S rRNA: 16S ribosomal ribonucleic acid
23S rRNA: 23S ribosomal ribonucleic acid
bp: Base pair
qPCR: Quantitative polymerase chain reaction
PCR: Polymerase chain reaction
