# Supplementary material for: Prevalence and phylogeny of Chlamydiae and hemotropic mycoplasma species in captive and free-living bats
Source: BMC Microbiol. 2020 Jun 26;20:182. doi: 10.1186/s12866-020-01872-x (PMC7318495; doi:10.1186/s12866-020-01872-x)
Supplement: Supplementary file 1 — Additional file 1: Supplementary Table 1. Reaction Mix compositions and cycling conditions of PCR methods used in this study. [file 12866_2020_1872_MOESM1_ESM.pdf]

Supplementary Table 1: Reaction Mix compositions and cycling conditions of PCR methods used in this study.

PCR test	Reaction Mix	Cycling conditions		
Chlam23S-qPCR	final volume: 25 µL	95°C	20 s	
	– 1X TaqMan Fast Universal PCR Master Mix (2X)[1]	95°C	3 s	45 x
	– 0.5 µM concentration of Ch23S-F and Ch23S-R each	60°C	30 s	
	– 0.2 µM concentration of Ch23S-p			
	– molecular-biology-grade water[1]			
	– 0.1 µM concentration of eGFP-1-F and eGFP-10-R each			
	– 1.0 µL eGFP-Hex			
	– 0.25 µL eGFP DNA template			
	– 2.5 µL sample DNA template			
16S-pan-qPCR	final volume: 20 µL	95°C	3 min	
	– iTaq Universal Probes Supermix with ROX[2]	95°C	15 s	40 x
	– 0.1 µM concentration of 16S-panCh-F and 16S-panCh-R	67°C	15 s	
	– 0.1 µM concentration of probe 16S-panCh	72°C	15 s	
	– molecular-biology-grade water[3]			
	– 5 µL of sample DNA template			
16S-IGF/IGR-PCR	final volume: 50 µL	95°C	5 min	
	– 1X PCR Puffer Roche (+ 20 mM $MgCl_2$)[4]	95°C	60 s	40 x
	– 0.5 mM $MgCl_2$[4]	65°C	60 s	
	– 0.2 mM dNTP[4]	72°C	90 s	
	– 0.3 µM concentration of 16S-IGF and 16S-IGR	72°C	10 min	
	– 0.02 U/µL FastStart™ Taq DNA Polymerase 5 U/µl[4]			
	– molecular-biology-grade water[1]			
	– 2 µL of sample DNA template			

16S-pan-PCR	final volume: 50 µL	95°C	5 min	
	– 1X AmpliTaq Gold 360 Master Mix[5]	95°C	1 min	40 x
	– 0.1 – 0.3 µM concentration of 16S-panCh-F and 16S-panCh-R	62°C	1 min	
	– molecular-biology-grade water[1]	72°C	1 min 30 s	
	– 3 µL of sample DNA template	72°C	10 min	
23SIG-PCR	final volume: 50 µL	95°C	5 min	
	– 1X AmpliTaq Gold 360 Master Mix[5]	95°C	1 min	40 x
	– 0.3 µM concentration of U23-F and 23SIG-R	62°C	1 min	
	– molecular-biology-grade water[1]	72°C	1 min 30 s	
	– 3 µL of sample DNA template	72°C	10 min	
Hemoplasma SYBR Green qPCR	final volume: 20 µL	95°C	3 min	
	– 1X Kapa SYBR Fast Mastermix (2X)[4]			
	– 1X Rox low (50X)[4]	95°C	3 s	40 x
	– 0.2 µM concentration of Mhae_sybr.359f, Mcocc_sybrF, Mhae_sybr.432r and Cmhae_Sybr.493r	60°C	30 s	
	– molecular-biology-grade water[1]	95°C	15 s	Melt Curve Stage
	– 5 µL sample DNA template	60°C	1 min	
		95°C	15 s	
		60°C	15 s	
Mhf-like qPCR	final volume: 25 µL	50°C	2 min	
	– 1X DNA qPCR Mastermix (2X)[6]	95°C	15 min	
	– 0.01 U/µL Uracil-N-Glycosylase (1 U/µL)[6]	95°C	10 s	45x
	– 0.9 µM concentration of Group_Mhf_fwd and Group_Mhf_rev	60°C	30 s	
	– 0.25 µM concentration of Group_Mhf_probe			
	– molecular-biology-grade water[1]			
	– 5 µL sample DNA template			

CMhm-like qPCR	final volume: 25 µL<br>– 1X DNA qPCR Mastermix (2X)[6]<br>– 0.01 U/µL Uracil-N-Glycosylase (1 U/µL)[6]<br>– 0.9 µM concentration of Group_CMhm_fwd and Group_CMhm_rev<br>– 0.25 µM concentration of Group_CMhm_probe<br>– molecular-biology-grade water[1]<br>– 5 µL sample DNA template	50°C<br>95°C<br>95°C<br>60°C	2 min<br>15 min<br>10 s<br>30 s	 <br>45x (10 s, 30 s)
HemMycop41/938-PCR	final volume: 25 µL<br>– 1X Phusion HF Buffer[1]<br>– 200 µM dNTP's (10 mM)[1]<br>– 1 U/µL Phusion Hot Start II High-Fidelity DNA Polymerase (2 U/µL)[1]<br>– 0.5 µM concentration of HemMycop16S-41s and HemMycop16S-938as<br>– molecular-biology-grade water[1]<br>– 5 µL sample DNA template	98°C<br>98°C<br>63°C<br>72°C<br>72°C	3 min<br>1 min<br>30 s<br>2 min<br>10 min	 45x (1 min, 30 s, 2 min)
HemMycop322/1420-PCR	final volume: 25 µL<br>– 1X Phusion HF Buffer[1]<br>– 200 µM dNTP's (10 mM)[1]<br>– 1 U/µL Phusion Hot Start II High-Fidelity DNA Polymerase (2 U/µL)[1]<br>– 0.5 µM concentration of HemMycop16S-322s and HemMycop16S-1420as<br>– molecular-biology-grade water[1]<br>– 5 µL sample DNA template	98°C<br>98°C<br>68°C<br>72°C<br>72°C	3 min<br>1 min<br>30 s<br>2 min<br>10 min	 45x (1 min, 30 s, 2 min)

[1] Thermo Fisher Scientific, Waltham, Massachusetts, USA
[2] Bio-Rad, Reinach, Switzerland
[3] Five Prime, Hilden, Germany
[4] Sigma-Aldrich Corp., St. Louis, Missouri, USA
[5] Life Technologies, Victoria, Australia
[6] Eurogentec, Seraing, Belgium
